# Prothrombotic markers in patients with acute myocardial infarction and left ventricular thrombus formation treated with pci and dual antiplatelet therapy

**DOI:** 10.1186/1477-9560-11-1

**Published:** 2013-01-11

**Authors:** Svein Solheim, Ingebjørg Seljeflot, Ketil Lunde, Vibeke Bratseth, Svend Aakhus, Kolbjørn Forfang, Harald Arnesen

**Affiliations:** 1 Center for Clinical Heart Research, Department of Cardiology, Oslo University Hospital, Ullevål, Postbox 4956, Nydalen, 0424, Oslo, Norway; 2 Faculty of Medicine, University in Oslo, Oslo, Norway; 3 Department of Cardiology, Oslo University Hospital, Rikshospitalet, Oslo, Norway

**Keywords:** Acute myocardial infarction, Haemostatic markers, Inflammation, Left ventricular thrombus formation

## Abstract

**Background:**

The aim of the present study was to compare circulating levels of selected prothrombotic markers in patients suffering acute myocardial infarction (AMI) with and without left ventricular (LV) thrombus.

**Methods:**

One hundred patients with AMI treated with PCI on the LAD and dual antiplatelet therapy were included. LV thrombus formation was detected by echocardiography and/or MRI in 15 patients. Fasting blood samples were drawn 4–5 days (baseline), 6–7 days, 8–9 days, 2–3 weeks and 3 months after the AMI for determination of haemostatic markers.

**Results:**

We found higher levels of soluble tissue factor (TF) and D-dimer in the LV thrombus group 4–5 days, 8–9 days and 3 months (only TF) after the AMI compared to the patients without thrombus formation (p<0.05). Patients with TF in the upper quartile at baseline had significantly higher risk for LV thrombus (OR 4.2; 95% CI 1.2 -14.5; p=0.02, adjusted for infarct size).

The levels of prothrombin fragment 1+2 (F1+2) and endogenous thrombin potential (ETP) were significantly lower in the thrombus group after 8–9 days (only ETP), 2–3 weeks and 3 months. The levels of plasminogen activator inhibitor 1 activity and tissue plasminogen activator antigen did not differ between the groups.

**Conclusion:**

In the acute phase of AMI, we found higher levels of TF and D-dimer in the LV thrombus group, indicating hypercoagulability of possible importance for the generation of mural thrombus. Lower levels of F1+2, ETP and D-dimer in the thrombus group late during follow-up are probably induced by the initiated anticoagulation therapy.

## Introduction

Left ventricular (LV) thrombus formation is a serious complication in patients with acute myocardial infarction and was reported in up to 60% of patients with acute anterior wall myocardial infarction before thrombolytic treatment was introduced [1,2]. In the percutaneous coronary intervention (PCI) era studies have shown that LV thrombus formation still occurs in patients with anterior wall myocardial infarction [3-6]. The exact mechanism for the development of mural thrombosis is not elucidated, but abnormal blood flow with stasis, endocardial injury with an inflammatory response and hypercoagulability are probably all involved.

The aim of the present study was to determine the circulating levels of prothrombotic markers in patients suffering acute anterior wall myocardial infarction with and without mural thrombosis, all treated with PCI and dual antiplatelet therapy. In addition, we wanted to examine any association between haemostatic variables and markers of inflammation.

## Materials and methods

### Materials

The study design, patients demographics and inclusion and exclusion criteria have been published in detail previously [5]. Briefly, one hundred patients with acute anterior wall ST-elevation myocardial infarction, both gender, age between 40 and 75 years were included at Ullevål University Hospital and Rikshospitalet University Hospital, Oslo, Norway. All the patients participated in the ASTAMI (Autologous Stem cell Transplantation in Acute Myocardial Infarction) trial with the culprit lesion located in the left anterior descending artery (LAD), proximal to the 2. diagonal branch, and were treated successfully with PCI and stent implantation within 2 – 12 hours from symptom start, in addition to dual antiplatelet therapy [7]. The inclusion criteria were peak creatine kinase (CK) MB above 3 times the upper reference level and hypo- or akinesia in more than 2 of 16 segments of the left ventricle determined by echocardiography. Patients with cardiogenic shock, previous Q-wave infarction and considerable co-morbidity with short life expectancy were excluded from the study. The investigation conformed with the principles outlined in the Declaration of Helsinki. The Regional Committee for Medical Research Ethics approved the study protocol, and written informed consent was obtained from all the patients. The present study is a substudy of the ASTAMI trial that is registered at http://www.clinicaltrials.gov, NCT 00199823.

### Methods

The included patients were randomized 1:1 to receive intracoronary injections of autologous mononuclear bone marrow cells (mBMC) or to a control group without any further interventions. The mBMC group was aspirated for 50 ml bone marrow from the iliac crest in local anesthesia 4 – 7 days after the acute PCI and the next day, a median of 6 days after the AMI, they received intracoronary injections of mBMC in the LAD. All the patients were treated with a loading dose of clopidogrel 300 mg and thereafter 75 mg daily in addition to an initial dose of aspirin 300 mg followed by 75 mg daily. Patients with LV thrombus were treated with low molecular weight heparin and further warfarin with a target international normalized ratio of 2.0 to 2.5 for at least 3–6 months. Fasting blood samples were drawn 4–5 days (baseline), 6–7 days, 8–9 days, 2–3 weeks and 3 months after the AMI for determination of circulating haemostatic markers and selected inflammatory mediators (only at baseline). In accordance with the study protocol a screening echocardiographic examination of all eligible patients was performed within the first 1–3 days after the acute PCI. Thereafter, echocardiograms were obtained by Vivid 7 scanner (GE Vingmed Ultrasound, Horten, Norway) of all the included patients 4.5 ± 1.1 days after the acute AMI and repeated after 3 months. Additional echocardiographic examinations were performed in between the predefined time points when clinically indicated. Magnetic resonance imaging (MRI) was performed with a 1.5-tesla scanner (Siemens, Germany) 18.8 ± 4.3 days after the acute PCI. Infarct size of the LAD area, in percent, was obtained by electrocardiogram-gated single photon emission computed tomography (SPECT) (GE Medical Systems with 4D-MSPECT software) 4.0 ± 1.4 days after the AMI.

### Enzyme immunoassays

Soluble tissue factor (TF) was measured by an enzyme-linked immunoassay (Imubind^®^ Tissue Factor, American Diagnostica Inc., US), prothrombin fragment 1+2 (F1+2) by an enzyme immunoassay (Enzygnost^®^F1+2, Siemens Healthcare Diagnostics, Marburg, Germany). Endogenous thrombin potential (ETP) was determined according to the manufacturer’s instruction (Thrombinoscope BV, Maastricht, The Netherlands) and the thrombin generation was measured on the Fluoroscan Ascent^®^ fluorometer (Thermo Fisher Scientific OY, Vantaa, Finland). A reagent of rTF and phospholipids in addition to a thrombin specific fluorogenic substrate (Z-Gly-Gly-Arg-AMC, Bachem, Bubendorf, Switzerland) in Hepes buffer containg CaCl_2,_ were added to the plasma prior to start to obtain a final concentration of 5 pM, 4 uM and 2.5 mM, respectively. In order to calculate the final results, plasma was analyzed along with a thrombin calibrator with known thrombin activity as a reference. The software program (Thrombinoscope BV, version 3.0.0.29) enabled the calculation of ETP. All the experiments were run in duplicates. D-dimer was measured by enzyme immunoassay (Asserachrom, D-di™, Stago Diagnostica, Asniere, France) and plasminogen activator inhibitor 1 (PAI-1) activity by a bio immunoassay (Trinilize PAI-1 activity, Trinity Biotech, plc, Bray, Co. Wicklow, Ireland). The levels of tissue plasminogen activator (tPA) antigen were determined by enzyme immunoassay (TintElize^®^tPA, Trinity Biotech, Jamestown, NY, US). Tumor necrosis factor α (TNFα) and interleukin 6 (IL-6) were measured by enzyme immunoassays obtained from R&D Systems Europe (Abingdon, Oxon, UK). C-Reactive protein (CRP) was determined by an enzyme-linked immunosorbent assay (DRG Instruments GmbH, Germany) (detection limit 0.1 mg/L). In our laboratory, the interassay coefficient of variation for TF was 4.4%; F1+2 7.8%; ETP 2.7%; D-dimer 6.5%; PAI-1 4.4%; tPA 3.5%; TNFα 8,5%; IL-6 10.7%; CRP<5%.

### Gene expression

Total RNA was extracted from PAXgene^®^ Blood RNA tubes in a subset of patients with (n=7) and without (n=10) LV thrombus. The PAXgene^®^ Blood RNA Kit (PreAnalytix, Qiagen GmbH, Germany), including an extra cleaning step (RNeasy^®^MinElute^®^ Cleanup Kit, Qiagen) was used. Total RNA was reversely transcribed in a total volume of 20 μl, using the Omniscript^®^ RT Kit (Qiagen), Oligos (dTs) and Rnase Inhibitor (Applied Biosystems, Foster City, CA, USA). TF mRNA levels were determined by real-time PCR on the ABI Prism 7900 HT Sequence Detection System, including the TaqMan^®^ Gene Expression TF Assay (Hs01076029_m1). The TF mRNA levels were normalized to β-2-microglobulin (Hs99999907_m1, Applied Biosystems) and fold expression (relative quantification using the ΔΔCt method) was determined in relation to a reference sample, as previously described [8].

### Statistical analysis

Variables are expressed as proportions, medians with 25,75 percentiles or means with standard deviation as appropriate. Differences between groups were analysed with the Mann–Whitney test for continuous variables. Categorical data were analyzed by the chi-square test. Friedman test was applied for testing the intragroup differences between several related samples and Wilcoxon test was used when Friedman test was significant, to assess within group changes from baseline to the subsequent time points. A trend analysis in quartiles of variables was performed by the chi square test to estimate the cut off levels in relation to LV thrombus. Logistic regression analyses with LV thrombus as the dependent variable were performed with adjustments for infarct size. Correlation analysis was done with Spearmans rho. All tests were two-sided, and p values <0.05 were considered statistically significant. PASW software package version 18.0 for Mac OS X was used for data analyses.

## Results

### General characteristics

Primary PCI was performed in 71 patients, facilitated PCI in 15 patients and rescue PCI in 14 patients without any differences between the groups. As previously described, we detected LV thrombus formation in 15 of 100 patients within the first 3 months, most of them diagnosed during the first week after the AMI [5]. LV thrombus formation was diagnosed by echocardiography in 13 patients and by MRI in 2 patients. No between group differences in baseline characteristics was found, except from significantly higher peak CK levels and larger infarct size assessed by SPECT in the patients with LV thrombus formation (Table 1).

**Table 1:** General characteristics of the patients with and without LV thrombus formation

**Variable**	**No (n=85)**	**Yes (n=15)**	**p-value**
Age (years)	57(50,64)	63(50,69)	0.18
Female	15(18%)	1(7%)	0.49
Current smoker	37(44%)	7(47%)	0.20
Hypertension	27(32%)	7(47%)	0.41
Hyperlipidemia	39(46%)	9(60%)	0.47
Diabetes mellitus	6(7%)	2(13%)	0.76
Previous myocardial infarction	4(5%)	0(0%)	0.89
Weight (kg)	84.0(74.5,92.5)	80(77.0,92.0)	0.99
Systolic blood pressure (mmHg)	130(120,144)	135(130,150)	0.33
Peak CK (ugr/L)	2197(1236,4182)	6128(4556,8061)	<0.01
Infarct size (% of LAD area by SPECT)	63.8(40.5,76.8)	82.5(71.3,93.1)	<0.01

Hypertension is defined as antihypertensive treatment or measured blood pressure above 140/90 mmHg. Hyperlipidemia is defined as baseline fasting total cholesterol above 5.5 mmol/L or previous use of cholesterol lowering medication. P-values refer to differences between the LV thrombus group and those without thrombus formation. CK; creatine kinase, LAD; the left anterior descending artery, SPECT; single photon emission computer tomography.

### Prothrombotic markers

#### Tissue factor

**Figure 1:**
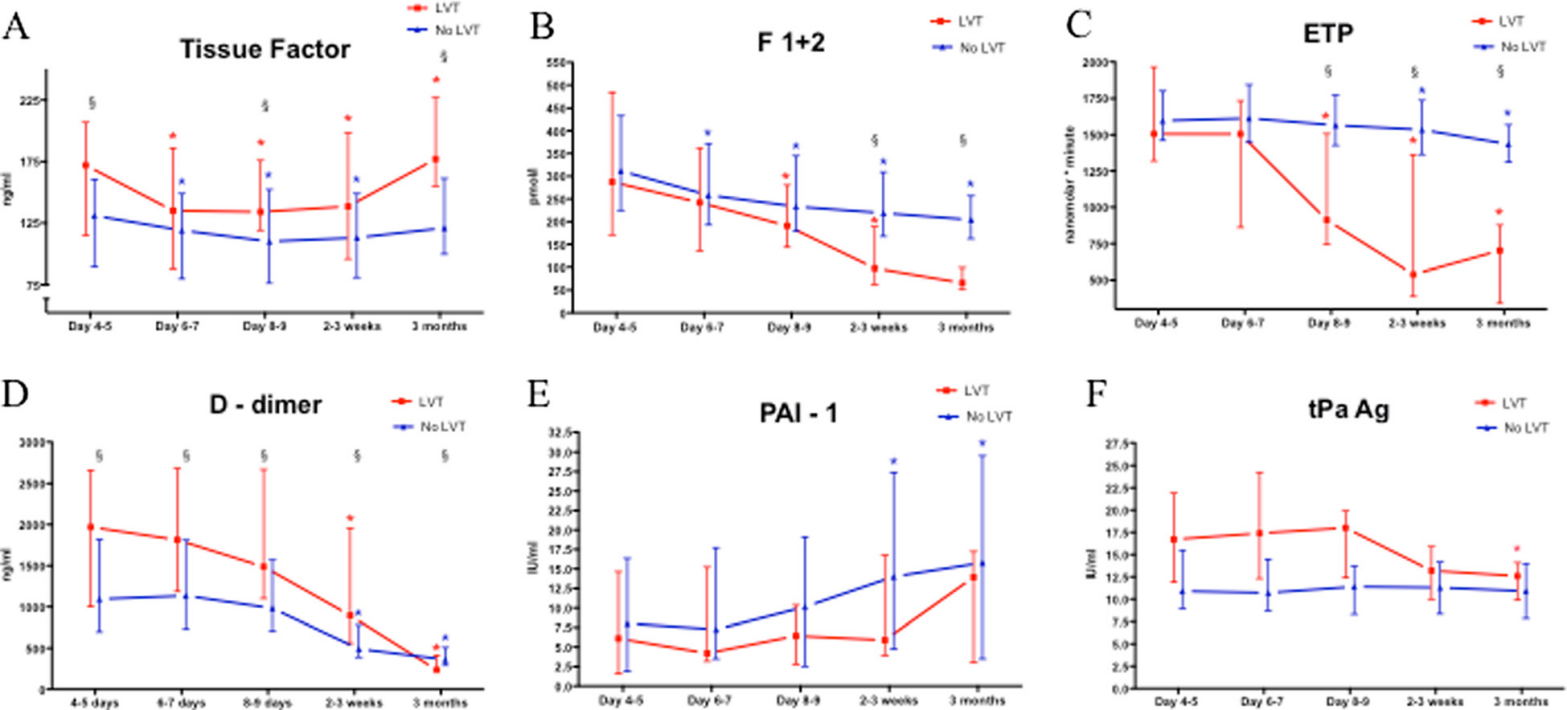
**Circulating levels of tissue factor, F1+2, D-dimer, PAI-1 activity, tPA antigen and ex vivo ETP measured 4–5 days, 6–7 days, 8–9 days, 2–3 weeks and 3 months after the AMI.** Median values with 25th, 75th percentiles are given. § refers to p-value < 0.05 for differences between the groups at the various time points, and * indicates p-value <0.05 for intragroup differences from day 4–5 to the subsequent time points. F1+2; prothrombin fragment 1+2, ETP; endogenous thrombin potential, PAI-1; plasminogen activator inhibitor 1 activity, tPA; tissue plasminogen activator antigen.

The levels of TF were significantly higher in the LV thrombus group 4–5 days, 8–9 days and 3 months after the AMI compared to the patients without thrombus formation (Figure 1, Panel A). In both groups, the TF levels declined significantly from day 4–5 to day 6–7, 8–9 and 2–3 weeks. Only in the thrombus group we found a small, but significant increase of TF levels from day 4–5 to 3 months. Trend analysis through quartiles of TF showed increasing numbers of LV thrombus with increasing levels of TF measured 4–5 days after the AMI. Patients with baseline TF levels in the upper quartile (≥167.8 ng/ml) had significantly higher risk for LV thrombus, with an odds ratio of 4.2 (95% confidence interval 1.5-14.4; p = 0.01), adjusted for infarct size assessed by SPECT 4–5 days after the AMI.

#### Tissue factor mRNA expression

**Figure 2:**
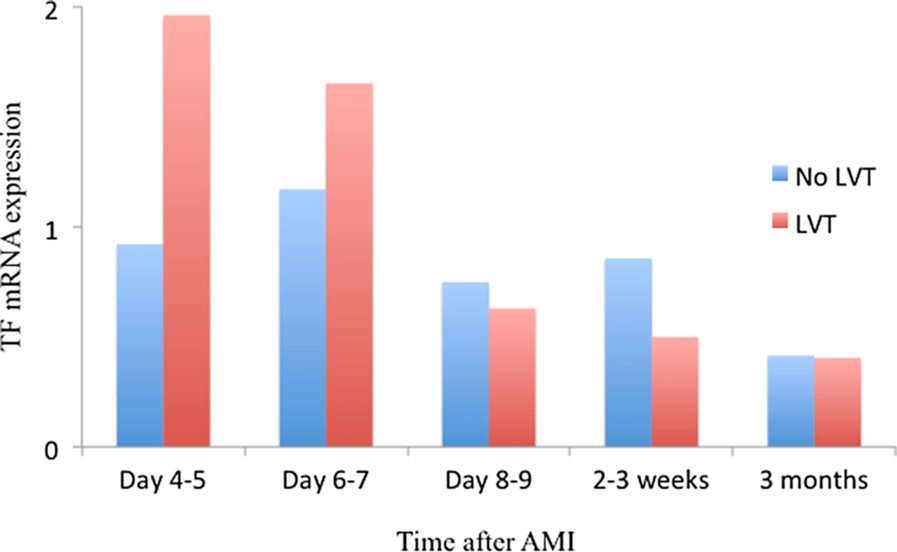
**The expression of mRNA TF in peripheral whole blood related to a reference sample at the various time points in the two groups.** TF; tissue factor.

The TF mRNA expressions were numerically higher in the LV thrombus group within the first 6–7 days after the AMI, however, not statistically significant (p>0.3 for all time points) (Figure 2). At later time points no between group differences were detected in the mRNA levels of TF.

#### Prothrombin fragment 1+2

A different pattern could be demonstrated for F1+2, with significantly lower levels in the thrombus group after 2–3 weeks and 3 months (p<0.01) (Figure 1, Panel B). In both groups, the F1+2 levels declined from 4–5 days to later time points.

#### Endogenous thrombin potential

A similar pattern as for F1+2 was found for ETP, with significantly lower levels in the thrombus group 8–9 days, 2–3 weeks and 3 months after the AMI and reduced levels in both groups from day 4–5 to day 8–9 and later on (Figure 1, Panel C).

#### D-dimer

The levels of D-dimer were significantly higher in the thrombus group at all time points except at 3 months where lower levels were obtained in the thrombus group (Figure 1, Panel D). In both groups the levels declined, but significantly only from day 4–5 to 2–3 weeks and 3 months. Patients with baseline D-dimer levels in the upper quartile (≥1960 ng/ml) had significantly higher risk for LV thrombus with an odds ratio of 3.8 (95% CI 1.1-13.0; p=0.03), adjusted for infarct size determined by SPECT 4–5 days after the AMI.

#### Tissue plasminogen activator antigen and plasminogen activator inhibitor 1 activity

No significant between group differences could be detected regarding the levels of tPA antigen and PAI-1 activity (Figure 1, Panel E and F). Only a minor reduction of the tPA levels from 4–5 days to 3 months could be demonstrated in the thrombus group and a small, but statistically significant increase of PAI-1 from day 4–5 to 2–3 weeks and 3 months in patients without thrombus formation.

### Circulating levels of selected inflammatory markers at baseline

The median levels of TNFα, IL-6 and CRP at baseline were numerically higher in the thrombus group versus those without LV thrombus, but not statistically significant (2.13 versus 1.89 pg/ml, p=0.57; 7.77 versus 7.63 pg/ml, p=0.49; 19.70 versus 16.39 mg/L, p=0.27).

### Correlation between TF and selected inflammatory mediators at baseline in the LV thrombus group

There was a significant correlation between the levels of TF and TNFα measured 4–5 days after the AMI with a coefficient of correlation of 0.55 (p=0.03), but not for IL-6 and CRP.

## Discussion

In the acute phase of AMI, we found significantly higher circulating levels of TF and D-dimer in the LV thrombus group, indicating hypercoagulability which might be of importance for the generation of mural thrombus. We have recently published that the patients with larger infarct size, evaluated with peak CK levels, had significantly increased risk for LV thrombus formation [5]. Larger infarct size is, undoubtedly, associated with increased risk for LV thrombus formation as shown in several studies [3,6,9-11]. In the present study we found, even after adjustment for infarct size, that high levels of TF and D-dimer, measured 4–5 days after the AMI, were associated with increased risk for LV thrombus with an odds ratio of 4.2 (1.5-14.4) and 3.8 (1.1-13.0), respectively. These findings may indicate that increased expression of TF plays a key role in the genesis of LV thrombus formation. Furthermore, the expression levels of TF mRNA in whole blood were higher in the thrombus group, although not statistically significant, probably due to limited numbers in these analyses. The higher D-dimer levels in the LV thrombus group are probably induced by increased thrombin generation and fibrin formation indicating increased procoagulant activity.

Interestingely, we also found a strong correlation between the levels of circulating TF and TNFα, that indicates an association between inflammatory responses and hypercoagulability in these patients. Several coagulant mediators are also proinflammatory [12]. Porreca et al. have shown a significant positive association between the levels of TF and CRP in hypercholesterolemic subjects [13]. Animal models have shown that TF may induce inflammatory artrithis with increased levels of IL-6 probably through a thrombin dependent pathway where both fibrin formation and platelet activation mediated by the receptor PAR-4, are essential [14]. On the other hand, inflammatory mediators themselves may also trigger coagulation. Kambas and coworkers suggest that TNFα is involved in the upregulation of TF in patients with acute respiratory distress syndrome [15]. These findings support our suggestion that co-activation of coagulation and inflammation plays a central in the formation of LV thrombus in the present population.

Late during follow up, we found lower levels of F1+2 and ETP in patients with LV thrombus compared to the non-thrombus group. This is probably because of ongoing anticoagulation therapy in these patients and thereby less thrombin generation. Higher levels of TF at 3 months in the LV thrombus group may be related to ongoing treatment with warfarin and less consumption of TF as previously reported [16]. In our study, we also found that several prothrombotic markers decreased after 2–3 weeks and 3 months in both groups indicating less hypercoagulability late during follow up after the AMI, which is in line with other studies [17]. Several clinical trials have also shown that increased levels of prothrombotic markers in AMI patients are associated with worse outcome [18-20]. In the present study, all patients were alive after 1 year with few non-fatal cardiovascular events, and any association between increased levels of prothrombotic markers and clinical outcome has therefore not been examined.

## Conclusion

Our findings indicate that hypercoagulability along with large infarct size and probably proinflammatory activity are key factors in the genesis of LV thrombus formation in patients with AMI treated with dual antiplatelet medication with limited effect on the coagulation system. The decline in procoagulant activity later on after the AMI, most pronounced in the LV thrombus group, is probably caused by ongoing anticoagulation therapy. Patients with large anterior wall myocardial infarction and high levels of TF and D-dimer have increased risk for LV thrombus formation and should be considered for anticoagulation therapy.

## Competing interests

The authors declare no competing interests.

## Authors’ contributions

SS conducted the study and was responsible for analysis and interpretation of the data, drafted and revised the manuscript. IS conducted the study, contributed to the interpretation of the results and revised the manuscript. KL contributed to the study protocol, acquired data and discussed the manuscript. VB has made substantial contributions to the analysis and interpretation of the data. SA and KF contributed to the study protocol, the interpretation of the results and discussed the manuscript. HA contributed to the study protocol, the interpretation of the results, discussed and revised the manuscript. All authors read and approved the final manuscript.
